# β-Naphthoflavone Activation of the Ah Receptor Alleviates Irradiation-Induced Intestinal Injury in Mice

**DOI:** 10.3390/antiox9121264

**Published:** 2020-12-12

**Authors:** Xiaoliang Zhou, Deguan Li, Wenqing Xu, Heng Zhang, Hao Wang, Gary H. Perdew

**Affiliations:** 1 Tianjin Key Laboratory of Molecular Nuclear Medicine, Institute of Radiation Medicine, Chinese Academy of Medical Sciences and Peking Union Medical College, Tianjin 300192, China; xuz221@psu.edu (X.Z.); lideguan@irm-cams.ac.cn (D.L.); xuwenqing@irm-cams.ac.cn (W.X.); 2 Department of Veterinary and Biomedical Sciences, The Center for Molecular Toxicology and Carcinogenesis, 309 LSB, The Pennsylvania State University, University Park, PA 16802, USA; 3 Department of Oncology, institute of Integrative of Oncology, Tianjin Union Medical Center, Tianjin 300191, China; 13602018589@163.com

**Keywords:** AHR, Ah receptor, β-naphthoflavone, oxidative stress, radioprotection, intestinal damage

## Abstract

Radiotherapy induced gastrointestinal syndrome results from the acute damage of intestinal stem cells, impaired crypts reconstruction, and subsequent breakdown of the mucosal barrier. The toxicity of ionizing radiation is associated with oxidative stress in the intestinal epithelial cells (IECs). Moreover, the rapid proliferation of IECs is a risk factor for radiation damage. β-naphthoflavone (BNF) is an agonist of the aryl hydrocarbon receptor (AhR) and possesses potential antioxidative activity. We investigated BNF radioprotection in IECs experiencing γ-ray exposure, contributed to mitigation of radiation enteritis. BNF significantly enhanced cell viability and suppressed cell apoptosis in an AhR activation-dependent manner. The mechanism of BNF reducing the IECs radiosensitivity was associated with cell cycle arrest and suppression of cell proliferation. In contrast, AhR antagonist CH-223191 significantly blocked BNF-induced cell cycle arrest. *Cyp1a1* mRNA levels are induced after irradiation in a dose-dependent manner, and CYP1A1 protein expression increased in the irradiated intestinal tract as well. BNF also reduces DNA strand breaks induced by irradiation. These studies demonstrate that BNF pretreatment prolonged median survival time of mice upon exposure to a lethal dose of radiation and alleviated irradiation-induced toxicity within the bowel.

## 1. Introduction

Irradiation induces gastrointestinal adverse effects in humans (~60% incidence rate) and is one of the primary limitations to delivering tumoricidal doses in abdominal radiation therapy. Although reversal of this toxicity is observed after radiotherapy has finished, 10% of the treated patients develop gastrointestinal syndrome, a disease characterized by intestinal cell death, which results in the destruction of the entire intestine and ultimately patient death [1]. Therefore, it is crucial to maintain intestinal homeostasis and intestinal integrity for clinical remission rate improvement. Currently, there is no clinically effective treatment for radiation enteritis, except some symptomatic treatment, such as, antibiotics, probiotics, feeding tube, and even surgical intervention.

The aryl hydrocarbon receptor (AhR) is a ligand-activated transcription factor that regulates the transcription of a wide range of genes, including some drug metabolizing enzymes (e.g., CYP1A1, CYP1B1). Recently, a number of reports have determined that the AhR is a modulator of immunological development, surveillance, and function within barrier tissues, such as the intestine [2,3]. Indeed, *Ahr^−/−^* mice are particularly susceptible to intestinal challenges, such as exposure to dextran sodium sulfate [4,5]. Moreover recent studies on oxidative stress reveal that interdependency of AhR and the Nrf2 pathway in gut epithelium enhanced barrier functions and reduced inflammation by upregulate epithelial tight junction proteins [6]. In previous in vitro studies, ionizing radiation lead to AhR activation, which also suggested a role of AhR in cellular oxidative stress in addition to the role of the AhR in xenobiotic metabolism [7].

β-naphthoflavone (BNF) is both a synthetic flavonoid and a non-carcinogenic AhR ligand, often used as a positive control for AhR activation [8]. Previous studies demonstrate that BNF upregulates the expression or activity of antioxidative enzymes, including glutathione peroxidase, quinone oxidoreductase-1, glutathione transferase and heme oxygenase-1 [9], and represses ROS-producing enzymes such as NADPH oxidase [10]. This suggests BNF potentially affects antioxidation [11]. In the present work, we show that BNF as an AhR agonist protects against oxidative DNA damage in the rat intestinal epithelial cell (IEC-6), while the ROS scavenging system is not involved. Additionally, we reveal that AhR ligands have a protective effect on total/local body irradiation induced intestinal injury in a mouse model.

## 2. Materials and Methods 

### 2.1. Cell Culture and Irradiation

The rat enterocyte cell line IEC-6 was cultured at 37 °C under a humidified atmosphere with 5% CO_2_ in DMEM-H medium (HyClone, Logan, UT, USA) supplemented with 0.01 mg/mL insulin, penicillin (100 units/mL), streptomycin (100 mg/mL; Gibco, Gaithersburg, MD, USA) and 5% fetal bovine serum (Gibco, USA). This line was chosen to be consistent with all of the studies being performed in rodent models. In addition, the cell line has phenotypic characteristics as undifferentiated epithelial cells in passage cultures and is considered to be derived from crypts in the small intestine. The culture medium was replaced by fresh medium with BNF (CAS: 6051-87-2; Sigma-Aldrich, St. Louis, MO, USA) or DMSO (final concentration 1%) before irradiation exposure. Cell irradiation was performed at the Institute of Radiation Medicine, Chinese Academy of Medical Sciences, with a Cs^137^ γ-radiation source (Atomic Energy of Canada Ltd.) at a dose rate of 1.02 Gy/min.

Suppression of AhR expression in IEC-6 cells was performed as previously described [12]. Briefly, the lentiviral pHBLV-U6-ZsGreen-Puro (Hanbio, Shanghai, China) was selected as the shuttle vector. Three interference sequence were designed and synthesized by Personalbio (China). Lentiviral shRNA vector targeting rat Aryl hydrocarbon receptor (rAhR) mRNA and control vector (vector shRNA) pHBLV-IRES-ZsGreen-PGK-puro (Figure S1) were purchased from Hanbio Biotechnology Co. Three interference sequence (Tables S1 and S2) were designed and synthesized by Personalbio (China). The linearized vectors, AhR templates and T4 DNA ligase (Fermentas) were mixed in T4 ligation buffer at 4 °C, overnight. The mixtures were transferred into E. coli DH5a cells. The positive colonies were isolated and subjected to DNA sequencing (BioSune, Shanghai, China). The purified rat-AhR-shRNA and three packaging plasmids were co-transfected in 293T cells. The transfection mixtures were incubated for 72 h at 37 °C, and the supernatant was collected and concentrated. IEC-6 cells were seeded in 6-well plate by 5 × 10^5^ density, and pHBLV-IRES-ZsGreen-PGK-puro (MOI = 20) was added and incubated for 24 h before the flash medium changed. The transfected cells were incubated for 48 h, then treated with 1 μg/mL puromycin for one week to complete the selection process.

To test the transfection efficiency of the rat-AhR-shRNA, the cells were monitored with a fluorescence microscope for green protein expression. The transfection efficiency was quantitated by qRT-PCR analysis. Total RNA was isolated using the Trizol Reagent (Invitrogen, Waltham, MA, USA), according to the manufacturer’s protocol. The primers utilized were: AhR (NM_001308254) forward 5′-CTACTACACGCCAGACCAGC-3′, AhR reverse 5′-CAACTGTGGAGGGAGTAGCG-3′ and GAPDH (NM_017008) forward 5′-GCTGGTGCTGAGTATGTCGT-3′, GAPDH reverse 5′- TCACAAACATGGGGGCATCA-3′.

IEC-6 cells were seeded in 24-well plates at a density of 5 × 10^4^ cells/well and grown overnight. Cells were transduced with the appropriate concentration of lentivirus (AhR shRNA or vector shRNA) at a multiplicity of infection of 20. After 48 h of transduction, puromycin (2 μg/mL) was added to the growth medium to select for virally integrated cells. Once cells reached confluency, they were trypsinized and transferred to a six-well culture dish and then to a T-75 culture dish. Puromycin was maintained in the medium during stable knockdown experiments to ensure maximal transgene expression.

### 2.2. Evaluation of BNF by Cytotoxicity and Radioprotection on Cell Lines

BNF cytotoxicity analysis and cell viability assay: BNF induced cytotoxicity was assessed by Cell Counting Kit-8 (CCK-8) in IEC-6 cells. Briefly, cells were seeded in 96-well culture plates with culture medium including vehicle (DMSO) or BNF (ranging from 10 nm to 100 μM) and were incubated for 24 h. After treatment, 10 μL of CCK-8 solution was added to each well, and the 96-well plate was continuously incubated at 37 ℃ for 1 h, then the OD value for each well was read at a wavelength of 450 nm to determine the cell viability using a microplate reader.

Cell viability assay was utilized to assess the effect of a non-cytotoxic dose of BNF or vehicle. Cells were treated with 1 μM BNF for 24 h prior to various doses of irradiation from 0 Gy to 8 Gy. After five days of radiation exposure, cell proliferation was monitored using CCK-8 solution, and cell viability was analyzed as a percentage of untreated cells.

Cell cycle analysis and cell apoptosis after irradiation: Six-wells plates were seeded with 1 × 10^6^ cells and treated with 10 μM CH-223191 (Sigma-Aldrich, USA) with 5 μM BNF, BNF alone, or a vehicle for 24 h, and were then harvested and washed with PBS. Cells were then treated with methanol and resuspended in PI-staining buffer (50 μL/mL PI, RNase A) for 15 min at 37 °C. Fluorescence intensity was analyzed by flow cytometer (BD Accuri™ C6 Plus, Franklin Lakes, NJ, USA).

For apoptotic cell analyses, 1 × 10^6^ cells were plated in six-well dishes and the next day treated with 1 μM BNF or a vehicle for 24 h prior to irradiation exposure at 0, 4, 8 Gy. After 24 h treatment, cells were harvested in PBS and analyzed with Annexin V Apoptosis Detection Kit with a flow cytometer (BD Accuri™ C6 Plus, USA).

Comet assay: Comet assays were adapted from the method described by Singh et al. [13]. The cells were exposed to irradiation from 0 to 12 Gy, after 24 h treatment with either vehicle or 1 μM BNF. A comet assay was conducted immediately following radiation exposure. Irradiated cells were washed with PBS and embedded in 0.5% low melting point agarose at a final concentration of 10^4^ cells/mL. Seventy-five microliters of the cellular suspension were then spread onto a frosted slide that had previously been covered with an initial layer of 100 μL of 0.5% normal melting point agarose. Slides were immersed in freshly prepared lysis solution (1% sodium sarcosinate, 2.5 M NaCl, 100 mM Na_2_EDTA, 10 mM Tris–HCl pH 10, 1% Triton X-100 and 10% DMSO) at 4 °C, overnight. The slides were then kept in a horizontal electrophoresis unit filled with fresh buffer (1 mM Na_2_EDTA, 300 mM NaOH, pH 13) for 20 min to allow DNA unwinding and exposure of alkali-labile sites, followed by electrophoresis for 20 min (20 V, 300 mA). The slides were then gently washed twice in neutralization buffer (0.4 M Tris-HCl, pH 7.5), and each slide was stained with ethidium bromide (20 μg/mL). All steps were performed under dim light to prevent nonspecific additional DNA breakage. The comet images were captured, and Comet Assay Software Project (CASP1.2.1, University of Wroclaw, Wroclaw, Poland) was employed to measure various comet parameters as previously described [14,15], and 50 cells per sample were randomly selected. The percentage tail DNA (% tail DNA) head DNA (% tail DNA) and OTM (Oliver Tail Moment) served as the indicators of DNA damage.

ROS level analysis on cells with irradiation exposure: The intracellular ROS levels were measured by flow cytometry with 2,7-dichlordihydrofluorescein diacetate (H_2_DCFDA, Invitrogen, USA), as the ROS probe after radiation exposure. IEC-6 cells were treated with 1 μM BNF or vehicle 24 h prior to irradiation with 0, 6, 12 Gy, followed by the addition of 5 μM H_2_DCFDA and 30 min incubation at 4 °C. Cells were washed twice with PBS and resuspended in FACS buffer (PBS + 0.1% FBS). Mean fluorescence intensity was measured on a flow cytometer (BD Accuri™ C6 Plus, USA).

Western blot analysis and real-time quantitative PCR: Cells were treated with 1 μM BNF or a vehicle for 24 h prior to radiation exposure, and were harvested after 5 h and homogenized in cell lysis buffer (Roche). Equal amounts of protein were separated by SDS-PAGE and electroblotted on PVDF membrane. The membrane was blocked for 1 h at room temperature with 5 % BSA in TBS (50 mM Tris–HCl, pH 7.5, 150 mM NaCl) containing 0.1 % Tween 20 and then incubated overnight at 4 ℃ with anti-AhR (1:100, Abcam, Cambridge, MA, USA), or β-actin antibody (1:1000, Santa Cruz, CA, USA), followed by incubation with HRP-conjugated Goat anti-Rabbit IgG and subsequent ECL detection (Cell Signaling, Danvers, MA, USA) essentially as described by the manufacturer. ECL exposure was scanned with a ChemiDoc MP Imaging System (Bio-Rad, Hercules, CA, USA).

The AhR shRNA or control shRNA IEC-6 cells were plated in 6-well plates at a density of 1 × 10^6^ cells, grown overnight and treated with 1 μM BNF or vehicle for 24 h before irradiation. Cells were harvested after 5 h irradiation, and total RNA was isolated from cells using TRIzol (Life Technologies). Total RNA (2 μg) was reverse transcribed to cDNA by cDNA synthesis kit (GoScript™ reverse transcription System, Promega). cDNA was pre-diluted to 2 ng/μL and 5 μL was used per RT reaction. The detection method used was based on Fast SYBR Green Master Mix reagent (Promega) chemistry and CFX Connect Real-Time PCR Detection System (Bio-Rad). Forward, and reverse sequences for Q-PCR were as follows: *Cyp1a1*(NM_012540), forward 5′-CACAGACAGCCTGATTGAGCA-3′, reverse 5′-GTGACTGTGTCAAACCCAGCTCCAAAGA-3′; GAPDH, forward 5′-GCTGGTGCTGAGTATGTCGT-3′, reverse 5′-TCACAAACATGGGGGCATCA-3′.

### 2.3. Animals and Irradiation

Animals, BNF exposure, and ionizing radiation: Male C57BL/6 mice (8 to 10 weeks) were provided by Beijing HFK Bioscience Co. Ltd. (Beijing, China). Animals were housed in the certified animal facility (Specific Pathogen Free level) at Institute of Radiation Medicine (IRM), Chinese Academy of Medical Sciences (CAMS). All procedures involving animals were reviewed and approved by the Animal Care and Use Committee of IRM (Permit Number 1638). Mice were treated with BNF in 200 μL of 90% corn oil/10% DMSO or vehicle. For the survival experiment, animals (*n* = 10 per group) were exposed to total body irradiation (TBI) at a single lethal dose of 7.2 Gy and then monitored for 30 days. With regard to the radiation challenge to the small intestine, mice (*n* = 6 per group) were exposed to a local body irradiation (LBI) dose of 15 Gy, and the small intestine was harvested for the histology parameter evaluation after 3.5 days. Two irradiation doses (15 Gy LBI and 7.2 Gy TBI) were chosen for in vivo experiments. In previous studies, 15 Gy local body irradiation exposure can induce Acute Radiation Syndrome (ARS) exhibiting the small intestinal symptoms without severe hematopoietic system damages, while the 7.2 Gy TBI was validated in the preliminary study as a lethal dose causing 80 to 100% mortality without treatment. All the ionizing radiation exposure was performed at the Institute of Radiation Medicine, Chinese Academy of Medical Sciences, with a Cs^137^ γ-radiation source (Atomic Energy of Canada Ltd.) at a dose rate of 1.02 Gy/min.

Histological Analysis: Mice were sacrificed, and jejunum was isolated after LBI 3.5 days, fixed overnight in 10% neutral formalin, and embedded in paraffin. Five-μm sections were prepared and stained with hematoxylin and eosin (H&E) according to standard protocols and observed under a light microscope (Olympus Corp, Tokyo, Japan).

Immunofluorescences analysis: The 5-μm sections of paraffin-embedded jejunum were processed for antigen retrieval using citrate buffer (pH 6.0) boiling for 20 min and then washed thoroughly with PBS. Samples were blocked with 5% fetal bovine serum for 30 min at room temperature and then incubated with anti-CYP1A1 antibody (1:1000 dilution, ThermoFisher Scientific, Waltham, MA, USA) overnight at 4 °C. After washing with PBS, slides were incubated with the secondary antibody conjugated with Alexa Fluor^®^488 (1:2000 dilution, Goat anti-Rabbit lgG, Thermofisher Scientific.) for 45 min at room temperature in a dark chamber. Nuclei were co-stained with DAPI (1:5000; Invitrogen) for imaging. Fluorescence was visualized with laser scanning confocal microscopy (Leica SPE, Germany), and quantitated with ImageJ software.

### 2.4. Statistical Analysis

To determine the appropriate sample size of irradiation experiments the following formula was used: Sample size = 2(Z_α/2_ + Z_β_)^2^ × P(1 − P)/(p_1_ − p_2_)^2^ and the values for each variable were; Z_α/2_ = 1.96 at type 1 error of 5%; Z_β_ = 0.842 at 80% power; p_1_ − p_2_ = D = difference in proportion of events in two groups; P = pooled prevalence. According the previous studies, the survival of irradiation group is about 10%, and drug treatment was expected to increase the survival to 75%. The sample size = 2 (1.96 + 0.842)^2^ × 0.475 (1 − 0.475)/(−0.65)^2^ = 9.3 and thus 10 mice/group were needed for this experiment. The results were expressed as the mean ± SD. Mouse survival curves were analyzed using the Kaplan-Meier method. Significant differences between experimental groups were evaluated using one or two-way analysis of variance (ANOVA) with repeated measures followed by post hoc comparisons with Tukey’s multiple paired comparison test. Significance thresholds of * *p* < 0.05, ** *p* < 0.01 and *** *p* < 0.001 were applied. All analyses were performed using GraphPad Prism software (San Diego, CA, USA).

## 3. Results

### 3.1. BNF Improves Cell Viability after Ionizing Radiation 

**Figure 1:**
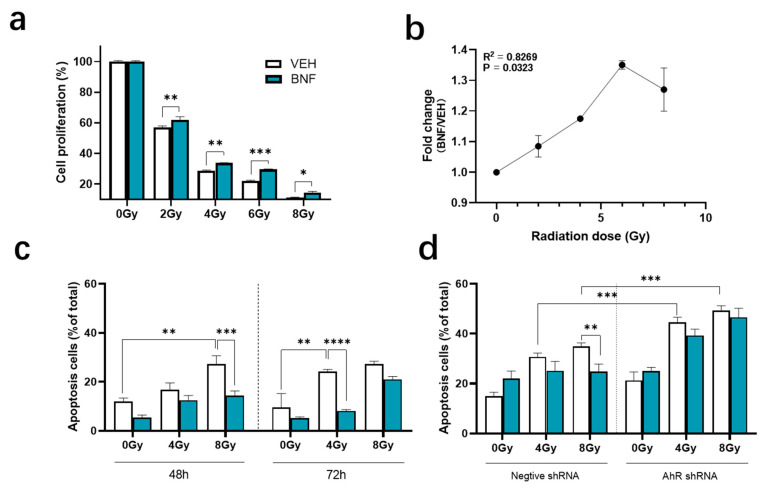
BNF (β-Naphthoflavone) protects cells from irradiation by regulating IEC-6 cell apoptosis. (**a**) Evaluation of radioprotection effect of BNF treatment on IEC-6 cells upon increasing radiation dose determined using a CCK-8 assay; (**b**) the cell proliferation fold change between BNF and vehicle treatment group with different radiation doses. (**c**) Cells treated with 1 μM BNF were tested for radiation-induced apoptosis after 48 h, 72 h radiation exposure; (**d**) AhR shRNA cells and control shRNA cells were tested for apoptosis after 48 h radiation exposure co-treated with either BNF or vehicle. Results are expressed as Mean ± SD and analyzed by either one- or two-way ANOVA (significance: * *p* < 0.05, ** *p* < 0.01, *** *p* < 0.001, **** *p* < 0.0001; vehicle group vs. BNF treatment group).

Toxicity of BNF was evaluated in IEC-6 cells with doses ranging from 0.01 to 100 μM. The IC_50_ of BNF is 14 ± 4 μM in IEC-6 cells, so 1 μM (near to IC_5_) was chosen as a non-toxic dose for cell experiments (Figure S2). Cell proliferation rate was dramatically suppressed to 57 ± 0.9%, 29 ± 0.4%, 22 ± 0.6% and 11 ± 0.1% from 2 to 8 Gy (Figure 1a), while treating cells with BNF prior to radiation significantly attenuated the loss of proliferative potential. The fold-change in proliferation rate correlated with radiation dose (R^2^ = 0.8269, *p* < 0.05), which increases from 1.0 to 1.4 at 0 to 8 Gy (Figure 1b). This pattern illustrates that BNF increases IEC-6 cells resistance to irradiation, while this protective effect reached peak value at 6 Gy and diminished as the radiation dose increased.

### 3.2. BNF Attenuates Cell Apoptosis after Radiation Exposure 

Apoptosis is a major mechanism of programmed cell death and can be induced by radiation, mediated by the lack of repair of DNA strand breaks. We tested whether BNF treatment could protect cells from apoptosis after exposure to radiation. The percentage of apoptotic cells increased from 5.5 ± 1.0% to 27.3 ± 3.4% (*p* < 0.01) after 48 h exposure to 8 Gy irradiation, no statistically significant difference was observed following BNF treatment in early apoptosis upon 0 and 4 Gy radiation exposure (Figure 1c). However, there was a significant decrease in percentage of apoptotic cells in the BNF-treated group at 8 Gy (14 ± 2.0% vs. 27.3 ± 3.4%, *p* < 0.001) exposure. After 72 h radiation exposure, the BNF-mediated suppression of cell apoptosis shifts from 8 to 4 Gy, (4 Gy irradiated vehicle group 24.3 ± 0.8% vs. BNF group 8.1 ± 0.6%, *p* < 0.0001), which suggests that this radioprotective effect of BNF shows a time-dependent pattern.

In order to confirm the AhR function in regulation of cell apoptosis, a shRNA targeting vector against the *Ahr* mRNA was developed, three shRNA targeting sequences were tested and sh2 was selected (Figure S3). The shRNA cells line was evaluated after 48 h radiation exposure (Figure 1d). The results show that the suppression of apoptosis induced by BNF is abolished with deficient AhR activity on AhR shRNA cells. Hence, there is no significant difference between BNF treated AhR shRNA cells at 0, 4, 8 Gy. Instead, the AhR shRNA cells with deficient AhR activity showed increased susceptibility to irradiation, leading to excessive cells apoptosis at 4 Gy (negative shRNA 307 ± 1.5% vs. AhR shRNA 446 ± 2.0%, *p* < 0.001) and 8 Gy (negative shRNA 35 ± 1.4 % vs. AhR shRNA 493 ± 2.0%, *p* < 0.001). These results reveal that cells with impaired AhR activity are more vulnerable to irradiation damage, and radioprotection of BNF is dependent on AhR activity.

**Figure 2:**
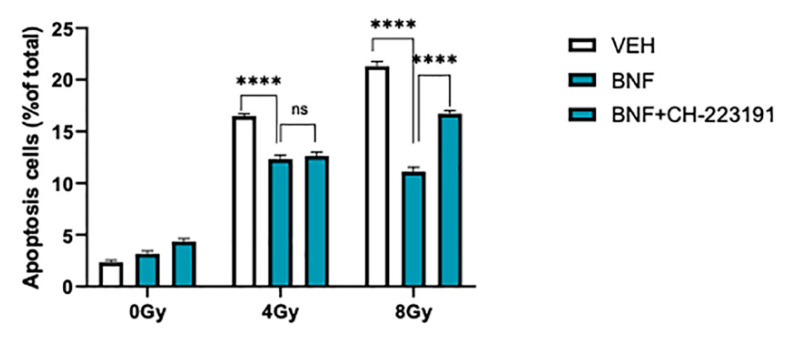
BNF protects cells from irradiation by regulating IEC-6 cells apoptosis. IEC-6 cells were tested for apoptosis after pre-treatment with 1 μM BNF or 1 μM BNF+ 1 μM CH232191. Results are expressed as Mean ± SD and analyzed by one-way ANOVA (Significance: **** *p* < 0.0001). The term “ns” is defined as not significant.

To further test whether BNF decreased irradiation-mediated apoptosis in an AhR-dependent manner IEC-6 cells were treated with BNF or BNF plus the AHR antagonist CH-232191 prior to irradiation (Figure 2). At 8 Gy exposure, BNF significantly protected cells (21.5 ± 0.4% vs. 11.4 ± 0.1%), while CH-232191 attenuated BNF-mediated decrease in apoptosis (16.7 ± 0.4% vs. 11.4 ± 0.1%).

### 3.3. BNF Induces Cell Cycle Arrest through AhR Activation

**Figure 3:**
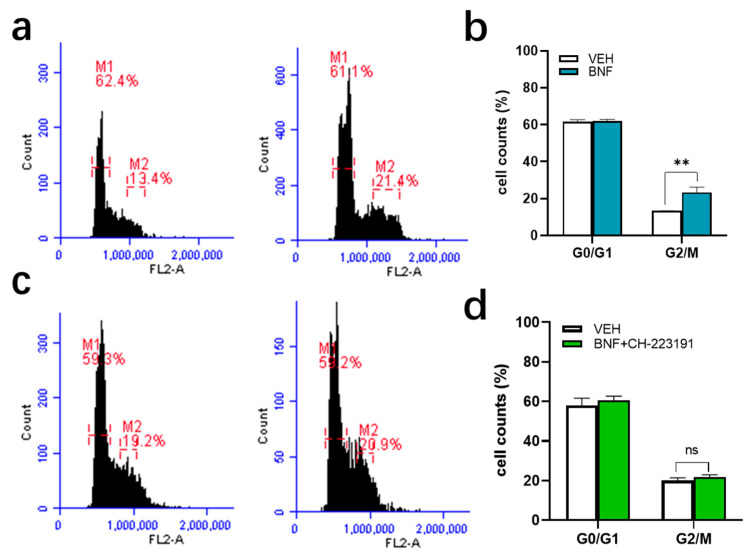
AhR activation in IEC-6 cells results in G2/M cell cycle arrest as determined by flow cytometry. (**a**,**b**) cells treated with BNF for 24 h or vehicle; (**c**,**d**) cells pretreated with AhR inhibitor CH-223191 (10 μM) and exposed to BNF or vehicle for 24 h. Results are expressed as Mean ± SD and analyzed by one-way ANOVA (Significance: ** *p* < 0.01). The term “ns” is defined as not significant.

To determine if the effect of BNF on IEC-6 cells is dependent on AhR, we utilized a specific AhR inhibitor CH-223191 (10 μM) to block AhR acitivation. First, we conducted a study in which cell cycle distribution was determined after cells were treated with BNF, which inhibits cell mitosis (Figure 3a,b). BNF-mediated AhR activaton led to a 75% increase in the percentage of cells in the G2/M phase of the cell cycle, which is 13 ± 0.1% in the vehicle treated group, compared with 23 ± 2.9% in the BNF treated cells. While the IEC-6 cells pretreated with CH-223191 abolished G2/M arrest induced by AhR activation, revealing the cell cycle arrest is AhR dependent (Figure 3c,d).

### 3.4. BNF Protected DNA from Radiation-Induced Strands Break

**Figure 4:**
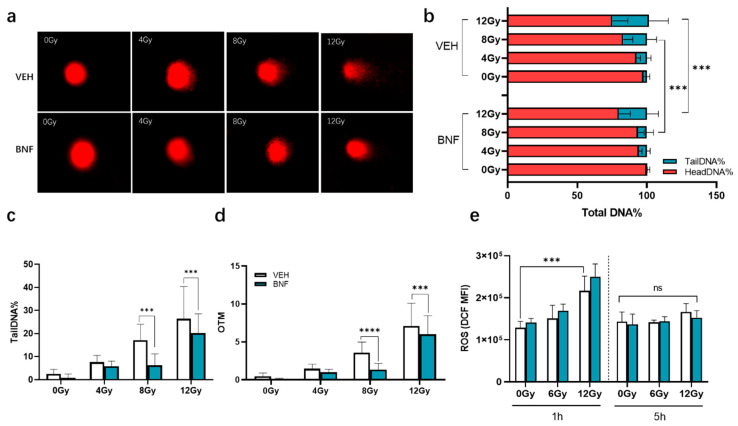
BNF diminishes the DNA strand breakage in IEC-6 cells exposed to irradiation. (**a**) Cell images of comet assay after various levels of radiation. (**b**–**d**) Percentage of head, tail DNA and olive tail moment based on comet assay. Percentage of DNA is quantitated by CASP1.2.1 software. (**e**) ROS level in irradiated IEC-6 cells. Cells were pretreated with 1 μM BNF or vehicle prior to irradiation and were harvested after irradiation exposure at 1 and 5 h and measured in a flow cytometer. Data represent the mean fluorescence intensity (*n* = 3) ± standard error (SD). Results were analyzed by one- or two-way ANOVA (Significance: *** *p* < 0.001, **** *p* < 0.0001). The term “ns” is defined as not significant.

Ionizing radiation of cells induces DNA strand breakage, which can be assessed by single cell gel electrophoresis (comet assay). DNA damage was determined as percentage of DNA in the tail (the most useful parameter of DNA damage). It exhibits a linear increase in percentage of DNA in the tail that corresponds to DNA damage, up to about 2.5 breaks per 10^9^ daltons. Significant DNA damage was observed in groups after irradiation with various doses of gamma-rays (Figure 4a). The TailDNA% is significantly enhanced in a dose-dependent manner increasing dose of irradiation. The range of DNA damage observed was from a minimum of 2.4 ± 2.1% at 0 Gy to the maximum of 26 ± 13% at the highest dose of 12 Gy at 1 h of post irradiation (Figure 4b). IEC-6 cells treated with 1 μM BNF prior to irradiation show significantly less DNA tailing at a dose of 8 and 12 Gy, suggesting activation of AhR could suppress DNA strand breakage or enhance repair caused by irradiation. The tailDNA% of vehicle group is 17 ± 6.9% and 26 ± 13% at 8 and 12 Gy, respectively, while that of BNF group is 6.2 ± 5 (*p* < 0.001) and 20 ± 8.3% (*p* < 0.001), respectively (Figure 4c). OTM shows a similar pattern as tailDNA%, which is 3.6 ± 1.4% (vehicle) vs. 1.3 ± 0.8 (BNF), *p* < 0.0001 and 7.1 ± 3.0 (vehicle) vs. 6.0 ± 2.5% (BNF) *p* < 0.0001, respectively, at 8 and 12 Gy (Figure 4d).

### 3.5. BNF does not Directly Contribute to ROS Scavenging

Excessive ROS accumulation induced by ionizing radiation directly causes DNA fragmentation. The intracellular ROS level was measured by DCF-DA in irradiated IEC-6 cells upon BNF treatment. The result shows that ROS increased significantly after irradiation 1.2-fold (6 Gy) and 1.7-fold (12 Gy, *p* < 0.001 vs. 0 Gy) compared with the control group and restored to normal basal level at 5 h. While 1 μM BNF pretreatment did not relieve the ROS accumulation on cells, ROS increased 1.2-fold and 1.8-fold compared to control at 6 and 12 Gy, respectively (Figure 4e).

### 3.6. BNF Activates AhR Activity in IEC-6 Cells after Irradiation 

**Figure 5:**
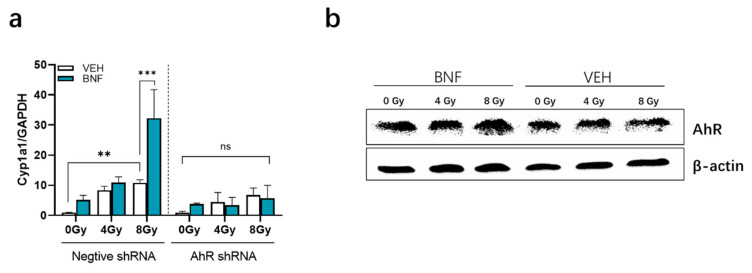
(**a**) *Cyp1a1* gene expression in the AhR shRNA and control vector cell lines treated with 1 μM BNF or vehicle prior to radiation exposure; (**b**) Western blot analysis of AhR protein expression in IEC-6 cells. Results are expressed as Mean ± SD and analyzed by one-way ANOVA (Significance: ** *p* < 0.01, *** *p* < 0.001). The term “ns” is defined as not significant.

AHR activation induces cytochrome P450 (CYP1A1) enzyme levels, which oxidize various AHR ligands, leading to their metabolic clearance and detoxification [16]. *Cyp1a1* expression is essentially only induced by an activated AHR, thus *Cyp1a1* can be used to assess AHR activation. Interestingly, *Cyp1a1* mRNA levels increased to 10 ± 1.0 in the control vector cell line 5 h after irradiation at 8 Gy, which is higher than the 0 Gy group by 1.0 ± 0.04 (*p* < 0.01) (Figure 5a). BNF exposure significantly drives *Cyp1a1* gene expression level to 32 ± 9.5 (*p* < 0.001). In contrast, there was a lack of significant induction of *Cyp1a1* mRNA by radiation exposure or BNF treatment in the AhR shRNA cells.

AhR protein expression after irradiation was evaluated by western blot analysis of irradiated IEC-6 cells. AhR expression in BNF-treated cells exposed was similar to counterparts in the vehicle group (Figure 5b).

### 3.7. BNF Improves Survival Time after TBI and Ameliorates Damage of Intestinal Morphology in Mice Exposed to LBI

**Figure 6:**
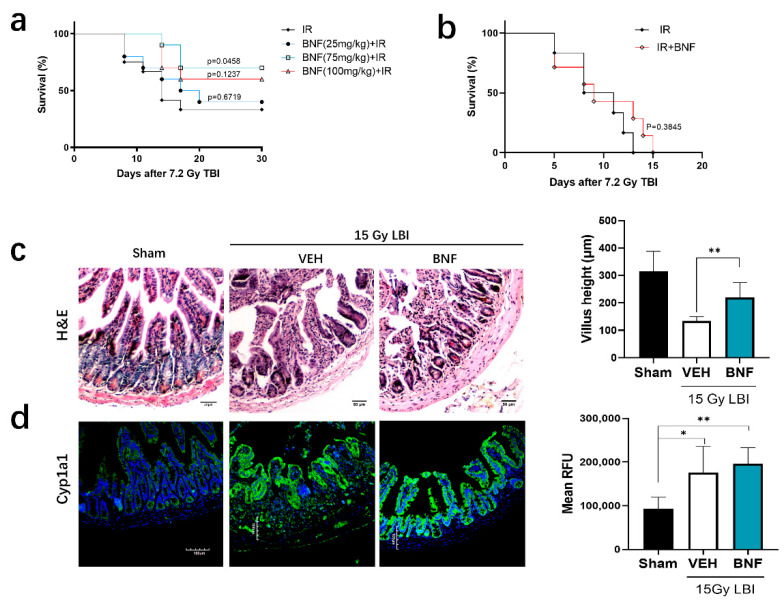
BNF protected mice from irradiation exposure. (**a**) Survival curves after 7.2 Gy of TBI. Mice (*n* = 10) were treated with different doses of BNF daily for seven days prior to 7.2 Gy TBI. (**b**) Mice (*n* = 6) were administered BNF at 75 mg/kg by gavage for seven days, on the seventh day mice were exposed to radiation, BNF treatment was continued for an additional five days, then monitored for 30 days. (**c**) H&E staining of jejunum cross-section and quantification of the villus height in jejunum. Mice were administered BNF (75 mg/kg) or vehicle by gavage daily for seven days prior to 15 Gy LBI irradiation.; (**d**) immunofluorescence of CYP1A1 protein levels in the jejunum of LBI irradiated mice and semi-quantification of fluorescence imaging of CYP1A1 expression. Results are expressed as Mean ± SD and analyzed by one-way ANOVA (Significance: * *p* < 0.05, ** *p* < 0.01).

BNF or vehicle (corn oil) was administered daily to C57/BL mice (*n* = 10) by gavage for seven days prior to administration of a TBI at a lethal dose of 7.2 Gy, followed by monitoring survival for 30 days. The results revealed that mice irradiated with a lethal dose started to die at one week, and most deaths occurred between days 8 to 16 in both groups. The survival rate of vehicle treated mice was 33.33% at day 30 (Figure 6a), while the BNF treated mice showed a higher survival rate of 40.0% (25 mg/kg/day), 70.0% (75 mg/kg/day), and 60.0% (100 mg/kg), compared to the control irradiated group. The median survival of the vehicle treated group was 14 days after exposure to lethal dose radiation, while the BNF (25 mg/kg) treated group median survival was 18.5 days. In contrast, the preventive treatment with BNF for seven days, followed by an additional administration for five days did not provide a benefit to the mice in terms of increased survival from lethal irradiation exposure, considering that the median survival was similar between the vehicle and BNF-treated mice (Figure 6b). Hence, BNF treatment prior to irradiation exhibited a protective effect for mice exposed to lethal dose irradiation, prolonging median survival. However, the observed decrease in body weight loss in the presence of BNF treatment was not significantly different from the vehicle treated mice (Figure S4).

Examination of the morphology of the small intestine revealed significant substructure changes in mice exposed to 15 Gy abdominopelvic irradiation after 3.5 days (Figure 6c). The following morphological changes were observed: disordered structure of jejunum, necrosis of a large number of epithelial cells to form necrotic villi with infiltration of large numbers of inflammatory cells, continuous crypt loss, shrinkage in crypt size, and shortening of the villi. Treatment with BNF (75 mg/kg/day) for seven days prior to radiation resulted in a greater number of normal crypts, intact villi, and general morphological improvement. The immunofluorescence imaging of CYP1A1 in the jejunum revealed increased expression after LBI irradiation, while mice pretreated with BNF also exhibited a similar overall level of CYP1A1 expression (Figure 6d).

## 4. Discussion

The higher turnover of IECs increases susceptibility of intestinal epithelium to ionizing radiation, which predisposes radiotherapy patients to suffer from the detrimental effects of gastrointestinal toxicity. Primarily, radiation induced enteritis syndrome can impair patient tolerance to the full course of treatment, and delayed GI symptoms occurring a few months or years after radiation injury can have a deleterious effect on patient quality of life [17]. Moreover, the resulting deficiency of nutrient absorption in the gastrointestinal tract causes severe weight loss and malnourishment, often leading to a poor prognosis [18]. In addition, radiation exposure generates excessive ROS causing DNA single-strand breaks and double-strand breaks along with intracellular alterations, resulting in apoptosis, autophagy and subsequent breakdown of the gut wall barrier. Increased cell death, specifically affecting mitotically active cells, delays epithelial repair, allowing penetration of antigens, bacterial products, and digestive enzymes. Clearly, therapeutic approaches are needed to mitigate radiation damage to the intestinal tract.

Maintenance of the overall number of intraepithelial lymphocytes within the gut requires expression of the AHR [19,20]. The AHR is also capable of inducing secretion of IL-22 in intraepithelial lymphocytes, which promotes stem cell regeneration [21]. In addition, the AHR participates directly within the IECs to modulate differentiation and protect against inflammatory damage [22]. The lack of AHR expression in IECs also leads to an impaired resolution of *C. rodentium* infection, underscoring the importance of AHR expression in barrier function and IEC homeostasis. The lack of AHR expression in intestinal organoids leads to enhanced IEC proliferation [23]. Interestingly, TCDD or FICZ inhibits mouse intestinal organoid growth, suggesting that excessive AHR activation affects proliferation of intestinal epithelial cells [24]. Thus, AHR activation influences both immune and epithelial cells within the intestinal tract, working together to improve barrier function and overall homeostasis.

The capacity of AhR ligands to mitigate disease severity in autoimmune, pathogenic, and chemical challenges are well documented [3,25,26]. Oxidative stress is induced by an alteration in the equilibrium between oxidative and antioxidative mechanisms and the influence of AhR activation on this process depends on organ specificity and/or cell type [27]. Importantly, dietary exogenous ligand(s) activation of the AhR upregulates *Cyp1a1* expression, yet inhibits ROS generation via the activation of an antioxidant master transcription factor, nuclear factor-erythroid 2-related factor-2 (NRF2) [6]. In contrast, the presence of “harmful” ligands, such as particulate matter containing polycyclic aromatic hydrocarbons, preferentially induces oxidative ROS generation even in the presence of NRF2 activation [28,29]. These studies suggest that the effects of AHR activation on oxidant stress is dependent on a number of factors, likely including metabolism and clearance differences of various AhR ligands, and thus is highly context specific.

The present study indicates that AhR activation is involved in the regulation of enterocyte radio sensitivity by coordinating IECs proliferation and cell cycle. Many studies have shown that AhR affects cell cycle progression through multiple mechanisms that are ligand- and cell-context dependent [30,31]. Considering cell viability evaluation data, the IC_50_ of BNF to IEC-6 cells is 13.77 ± 3.632 μM, which is significantly less than the concentration utilized in intestinal cancer cell studies [32]. Although the IEC-6 cells exposed to BNF 1 μM (IC_5_) have no obvious viability change, cells exhibited resistance to irradiation. The results also revealed a correlation between radiation dose and fold-change in proliferation upon exposure to BNF relative to control cells. Whether cells proliferate or remain quiescent depends on signaling pathways that link information influencing the G1 phase of the cell cycle. For example, both mitogenic and antiproliferative signals exert their effects on cell proliferation through the transcriptional regulation and ubiquitin-dependent degradation of cyclins and CDK inhibitors [33]. Indeed, the AhR is capable of directly regulating the cell cycle inhibitor p21 [34,35]. However, whether p21 is regulated in IECs or intestinal stem cells leading to an inhibition of proliferation has not been established. Under normal conditions, activation of AhR causes its translocation to the nucleus where it functions as an environmental checkpoint in cooperation with RB/E2F, inhibiting cyclin D, E/cdk-dependent RB phosphorylation, promoting repression of S-phase specific genes and causing cell cycle arrest. While under abnormal conditions of DNA damage, the AhR is activated and interacts with E2F-1 inhibiting apoptotic signaling, leading to an antiapoptotic response [36]. AhR activation induces cell cycle arrest in the G2/M stage, which decreases cell sensitivity to ROS. Clearly, additional studies are needed to better understand the mechanism(s) of AHR-mediated regulation of cell proliferation.

Our results reveal that BNF treatment can significantly mitigate apoptosis stimulated by irradiation exposure in IEC-6 cells. In contrast, the regulation is impaired in the cells with attenuated AhR activity, which demonstrates that the impact of apoptosis is AhR activity dependent. Use of the comet assay is another important parameter to evaluate the radiation sensitivity in vitro and in vivo, as ionizing radiation induced double strand breaks is the most lethal form of DNA damage and a primary cause of cell death [37]. In our study, BNF protection of IECs from DNA damage is dose-dependent and most likely associated with the antioxidative AhR pathway activation as a defense mechanism. These results are consistent with studies from others demonstrating that indolo[3,2*b*]carbazole can protect several cell lines (e.g., Caco-2 cells) from oxidative DNA damage [38]. In order to verify the antioxidative defense mechanism involved in the radioprotection of AhR agonist, we analyzed the intracellular ROS levels after irradiation using oxidized H_2_DCFDA by flow cytometry. Ionizing radiation stimulated ROS accumulation in a dose-dependent manner, however, the BNF treatment did not suppress ROS levels compared with vehicle counterparts. Therefore, the primary mechanism of BNF radioprotection of IECs may be associated with its influence on the cell cycle or cell survival signals. To better understand AhR activation potential after irradiation exposure, AhR protein levels were analyzed in the presence or absence of BNF. AhR protein expression was similar in the irradiated IECs, before and after BNF exposure. Nevertheless, ionizing radiation induced ROS may contribute to increased AhR expression directly [39], while a previous study suggested that irradiation can lead to AhR protein stabilization and translocation into the nucleus [7]. Perhaps the most intriguing result observed here in the in vivo studies is the increase in CYP1A1 expression in the jejunum after radiation exposure, which might suggest that upon radiation, an AhR ligand is produced. While speculative, it is possible that an AhR ligand(s) is produced through a free radical or oxidative mechanism, perhaps similar to that observed with tryptophan in the presence of oxidant stress [40]. Nevertheless, the putative presence of AhR ligand after radiation exposure may contribute to an inhibition in cell proliferation.

In our study examining BNF radioprotection in vivo, results show that BNF exhibited a preventative phenotype leading to prolonged survival time of mice exposed with lethal dose irradiation. The histological observation shows alleviated intestinal toxicity, contributing to intestinal function recovery. However, when we treated mice with BNF for five days after irradiation to evaluate its therapeutic effect, there was no obvious beneficial effect observed. This result is consistent with excessive suppression of IECs proliferation after irradiation, which likely would cause impairment of crypt regeneration, which is crucial for the maintenance of intestinal tract integrity [38].

In summary, our study demonstrates that BNF mitigates irradiation-induced intestinal system injury in mice exposed with TBI or LBI, and this protection is due to AhR activation associated with other antioxidative defense mechanism(s). Additionally, AhR activation can induce IECs cell cycle arrest at G2/M stage, consequently, reducing IECs sensitivity to irradiation. Hence, it is essential to maintain proper AhR activity in IECs experiencing radiotherapy; specifically, an AhR ligand-rich diet may be considered as potentially supportive care to benefit tumor therapies in the clinic [41]. However, the appropriate level of exposure to AHR for optimal results should be further examined. In addition, more research is necessary to further clarify the detailed molecular mechanisms of AhR pathway activation and the relationship between AhR activity and radiotherapy progression.

## Supplementary Materials

The following are available online at https://www.mdpi.com/2076-3921/9/12/1264/s1, Figure S1: Plasmid map of pHBLV-U6-ZsGreen-Puro lentiviral vector; Figure S2: BNF cytotoxicity analysis; Figure S3: The relative expression of AhR gene in transfected IEC-6 cells; Figure S4: *Lack of* BNF impact on weight loss in mice after lethal irradiation exposure. Table S1: Interference sequences targeting for rat AhR gene; Table S2: Oligonucleotides sequences used in this study.
