# Supplementary material for: β-Naphthoflavone Activation of the Ah Receptor Alleviates Irradiation-Induced Intestinal Injury in Mice
Source: Antioxidants (Basel). 2020 Dec 12;9(12):1264. doi: 10.3390/antiox9121264 (PMC7763649; doi:10.3390/antiox9121264)
Supplement: Supplementary file 1 [file antioxidants-09-01264-s001.pdf]

Supplements: Supplemental Data Tables, and Figures.

S.1 Plasmid map of pHBLV-U6-ZsGreen-Puro lentiviral vector.

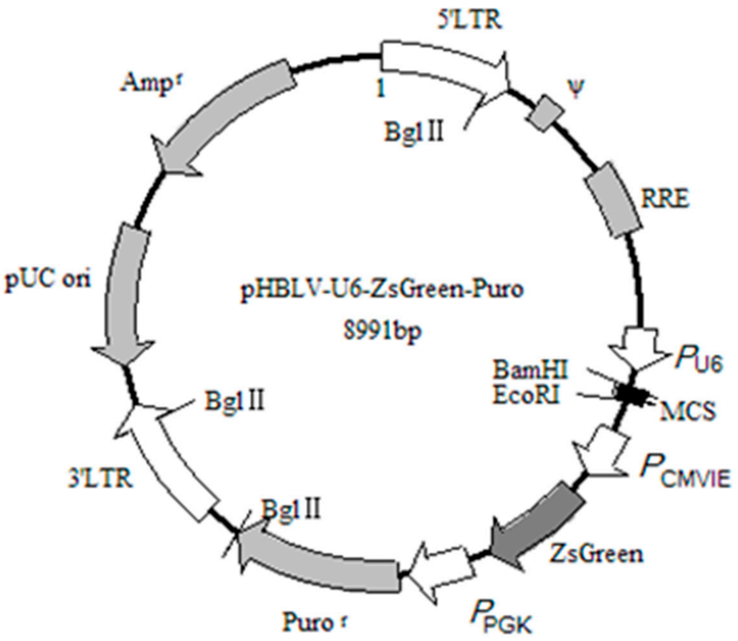

Fig. S1. Plasmid map of pHBLV-U6-ZsGreen-Puro lentiviral vector.

Table S1. Interference sequences targeting for rat AhR gene

Gene	Sequence
Rat-AhR shRNA1	GAGAGTTCTTGTTACAGGCGCTGAA
Rat-AhR shRNA2	GGATGAAGAAGGACGCGAACA
Rat-AhR shRNA3	CAGAACATTCCAAGGGTACACAGTT

Table S2. Oligonucleotides sequences used in this study.

Gene		Sequence(5'-3')
shRNA1	F	GATCCGAGAGTTCTTGTTACAGGCGCTGAATTCAAGAGATTCAGCGCCTGTAACAAGAACTCTCTTTTTTC
	R	AATTGAAAAAAGAGAGTTCTTGTTACAGGCGCTGAATCTCTTGAATTCAGCGCCTGTAACAAGAACTCTCG
shRN	F	GATCCGGATGAAGAAGGACGCGAACATTCAAGAGATGTTCGCGTCCTTCTTC

A1		ATCCTTTTTTC
	R	AATTGAAAAAAGGATGAAGAAGGACGCGAACATCTCTTGAATGTTCGCGTCC TTCTTCATCCG
shRN A1	F	GATCCGCAGAACATTCCAAGGGTACACAGTTTTCAAGAGAAACTGTGTACCCT TGGAATGTTCTGTTTTTTC
	R	AATTGAAAAAACAGAACATTCCAAGGGTACACAGTTTCTCTTGAAAACTGTGT ACCCTTGGAATGTTCTGCG

S.2 *BNF cytotoxicity analysis*

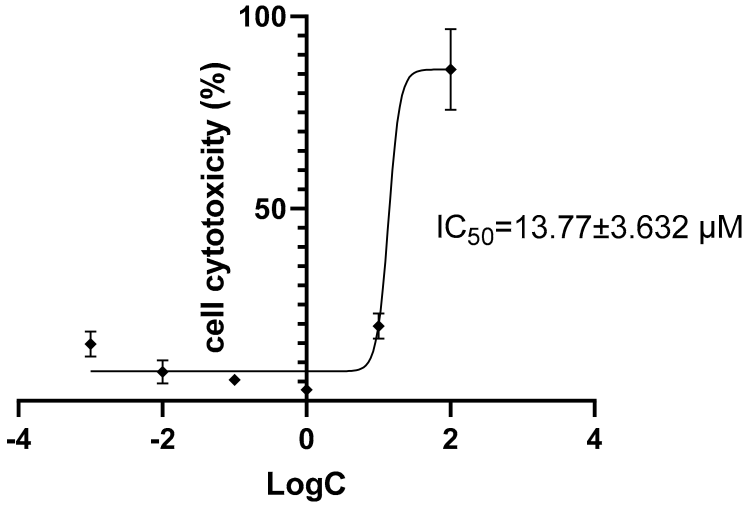

Fig. S2. Cytotoxicity of BNF on IEC-6 was measured by CCK-8 assay; Data present the mean cell viability inhibition (n=3) ±standard error (SD).

S3. Selection of the most efficient AhR shRNA2 vector.

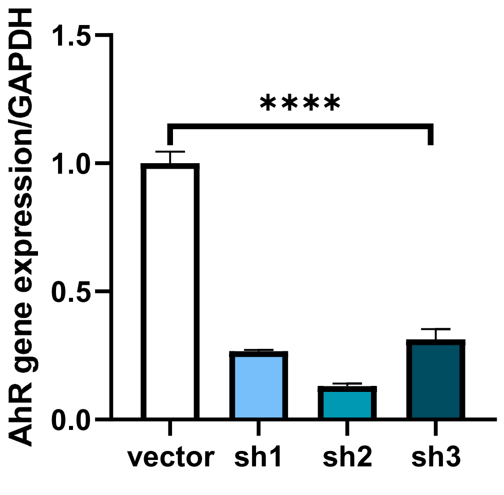

Fig. S3. The relative expression of AhR gene in transfected IEC-6 cells. AhR gene expression in the transfected cells decreased significantly, and the AhR shRNA2 selected in further experiment is the most effective knock-down cell line by 87% reduction of AhR gene expression. Data present the mean relative AhR gene expression (n=3)±standard error (SD).

S.4 Lack of *BNF impact on weight loss in mice after lethal irradiation exposure.*

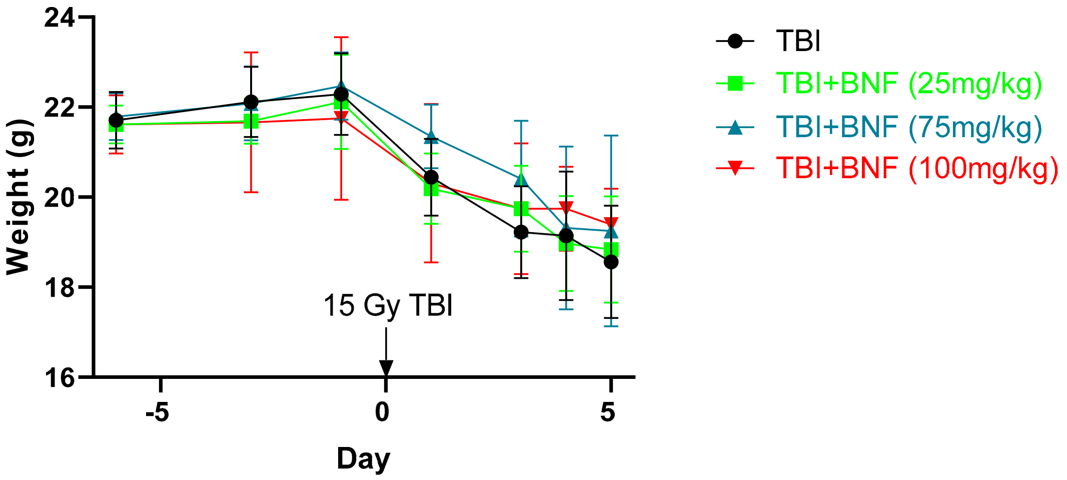

Fig. S4. BNF impact on body weight in the irradiated mice. C57/BL mice (n=6) were treated with 25, 75, 100 mg/kg BNF by gavage for a total of 7 days; mice were irradiated with by 7.2 Gy TBI on day 3. Mouse weight was monitored for 11 days. Data present the mean weight ± standard error (SD).
